# Impact of New Scatter Correction Strategies on High-Resolution Research Tomograph Brain PET Studies

**DOI:** 10.1007/s11307-015-0921-x

**Published:** 2016-01-04

**Authors:** Syahir Mansor, Ronald Boellaard, Marc C. Huisman, Bart N. M. van Berckel, Robert C. Schuit, Albert D. Windhorst, Adriaan A. Lammertsma, Floris H. P. van Velden

**Affiliations:** Department of Radiology and Nuclear Medicine, VU University Medical Center, PO Box 7057, 1007 MB Amsterdam, The Netherlands; Department of Radiology, Leiden University Medical Center, Leiden, The Netherlands; Department of Nuclear Medicine and Molecular Imaging, University Medical Centre Groningen, Groningen, The Netherlands

**Keywords:** Positron emission tomography (PET), High-resolution research tomograph (HRRT), Scatter correction, Scatter scaling factor, Patient motion, Single scatter simulation, ACF-margin

## Abstract

**Purpose:**

The aim of this study is to evaluate the impact of different scatter correction strategies on quantification of high-resolution research tomograph (HRRT) data for three tracers covering a wide range in kinetic profiles.

**Procedures:**

Healthy subjects received dynamic HRRT scans using either (*R*)-[^11^C]verapamil (*n* = 5), [^11^C]raclopride (*n* = 5) or [^11^C]flumazenil (*n* = 5). To reduce the effects of patient motion on scatter scaling factors, a margin in the attenuation correction factor (ACF) sinogram was applied prior to 2D or 3D single scatter simulation (SSS).

**Results:**

Some (*R*)-[^11^C]verapamil studies showed prominent artefacts that disappeared with an ACF-margin of 10 mm or more. Use of 3D SSS for (*R*)-[^11^C]verapamil showed a statistically significant increase in volume of distribution compared with 2D SSS (*p* < 0.05), but not for [^11^C]raclopride and [^11^C]flumazenil studies (*p* > 0.05).

**Conclusions:**

When there is a patient motion-induced mismatch between transmission and emission scans, applying an ACF-margin resulted in more reliable scatter scaling factors but did not change (and/or deteriorate) quantification.

## Introduction

The high-resolution research tomograph (HRRT; CTI/Siemens, Knoxville, TN, USA) is a dedicated human brain positron emission tomography (PET) scanner that consists of a smaller bore (46.9 cm) and a longer axial field of view (FOV; 25.2 cm) than current commercial PET scanners [1]. It has a spatial resolution of 2 to 3 mm full width at half maximum (FWHM). Due to its higher spatial resolution, it has the potential to provide a more accurate reflection of true radioactivity distributions compared with most clinically available PET/CT systems. In recent years, there has been considerable progress in developing more advanced data correction and reconstruction for the HRRT [2–5]. However, the HRRT still suffers from inaccuracies in scatter correction [6, 7]. The number of scattered events can amount to 56 % of the total number of events [8]. Frequently, scatter is estimated using a 2D single scatter simulation (SSS) algorithm that originally has been developed for whole-body PET imaging [9]. SSS is a computationally efficient method that only models the contribution of single scatter events (*i.e.* those that result from annihilation photon pairs in which a photon has been scattered only once before being detected) and typically uses tail fitting to match modelled scatter data with measured data. 2D SSS only takes scatter coincidences in non-oblique imaging planes into account. Later, 2D SSS was extended to 3D SSS to incorporate scatter coincidences in oblique planes [2]. A preliminary study by Hong *et al*. [10] indicated that, compared with its 3D variant, 2D SSS overestimated scatter for brain regions with low levels of tracer uptake (up to 25 % in-plane difference). A phantom study confirmed that 2D SSS shows bias (5 % in the centre of a cylindrical phantom) that disappears with 3D SSS [2]. However, the impact of 3D SSS on kinetic parameters derived from dynamic brain studies has not been assessed yet.

**Fig. 1:**
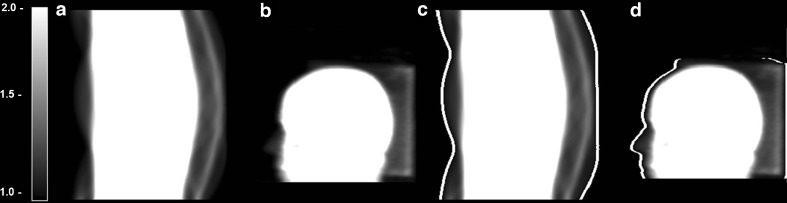
**a**, **c** ACF sinogram and **b**, **d** projection of a typical subject, **c**, **d** with or **a**, **b** without an applied ACF-margin of four voxels.

As reported by Anton-Rodriguez *et al*. [6], HRRT brain studies using (*R*)-[^11^C]verapamil, a substrate of the efflux transporter P-glycoprotein (P-gp), may show prominent artefacts in the lower part of the brain that are caused by patient motion. This patient motion leads to a misalignment between transmission and emission data, resulting in incorrect tail fitting that, in turn, causes an overestimation of scatter scaling factors. This misalignment between emission and transmission data can be corrected for by either using a motion tracking device [11] or image-based motion correction methods [12]. Use of a motion tracking device, however, requires additional hardware and processing software that are not readily available at all imaging sites. If the magnitude of patient motion is small enough, simpler methods (without the need for additional hardware) may suffice in order to avoid inaccuracies due to overestimation of scatter scaling factors. The most recent version of the HRRT software (version 1.3) allows users to specify an additional margin (in voxels) in the attenuation correction factor (ACF) sinogram (so-called ACF-margin, illustrated in Fig. 1). This ACF-margin is used only for scatter scaling estimation and assures that this estimation is less sensitive to a mismatch between transmission and emission data [6]. A recent study [7] showed that this ACF-margin did not result in visible artefacts in (*R*)-[^11^C]verapamil HRRT brain studies. Nevertheless, effects on quantification and the optimal ACF-margin enabling compensation for most of the clinically observed patient motions in HRRT brain studies still need to be determined.

The aim of this study was to evaluate the impact of an ACF-margin in combination with both 3D and 2D SSS on quantification of HRRT data for three tracers covering a wide range in kinetic profiles, *i.e*. (*R*)-[^11^C]verapamil, [^11^C]raclopride, a dopamine D_2_ receptor antagonist, and [^11^C]flumazenil, an antagonist of the benzodiazepine site of the γ-aminobutyric acid (GABA_A_)-receptor.

## Materials and Methods

### Subjects and Data Acquisition

A total of 15 healthy subjects received dynamic HRRT scans using either (*R*)-[^11^C]verapamil (*n* = 5), [^11^C]raclopride (*n* = 5) or [^11^C]flumazenil (*n* = 5). The mean age (±SD) of the subjects was 61 ± 4, 27 ± 3 and 53 ± 16 years for (*R*)-[^11^C]verapamil, [^11^C]raclopride and [^11^C]flumazenil, respectively. All studies were approved by the Medical Ethics Review Committee of the VU University Medical Center, and all subjects gave written informed consent prior to scanning. One week prior to the PET scan, a structural T1-weighted magnetic resonance (MR) image was obtained for each subject using a SONATA 1.5T MR scanner (Siemens Medical Solutions, Erlangen, Germany), which was used for co-registration and volumes of interest (VOI) definition purposes. For PET, first, a transmission scan was acquired using a ^137^Cs point source, which was used for attenuation correction of the subsequent emission scan. Next, a 60-min emission scan was started in 3D acquisition mode simultaneously with an intravenous injection of the tracer. To limit head movement, a head immobilization device was used and subjects were instructed to refrain from moving their head during the entire scan procedure. During the emission scan, arterial blood was sampled continuously using an on-line blood sampling device [13]. At set times (5, 10, 15, 20, 30, 40 and 60 min p.i.), continuous sampling was interrupted briefly to collect manual blood samples. More details on the study protocols can be found elsewhere [7, 14].

### Image Reconstruction and Data Processing

Acquired list-mode PET emission data were histogrammed into 20 different time frames (1 × 15, 3 × 5, 3 × 10, 2 × 30, 3 × 60, 2 × 150, 2 × 300 and 4 × 600 s) for all [^11^C]raclopride and (*R*)-[^11^C]verapamil studies and into 16 time frames (4 × 15, 4 × 60, 2 × 150, 2 × 300 and 4 × 600 s) for all [^11^C]flumazenil studies. Sinograms were normalized and corrected for scatter and random events, attenuation, decay and dead time, and reconstructed using a resolution-modelled ordinary Poisson ordered-subset expectation maximization algorithm [15] using 12 iterations and 16 subsets [10]. Reconstructed images had a matrix size of 256 × 256 × 207 voxels with an isotropic voxel volume of 1.22 mm^3^. The default parameters for resolution modelling were used [15]. Attenuation correction was performed using a transmission total variation regularization algorithm as described previously [3]. After reconstruction, no plane efficiency correction factors were applied to the reconstructed images to correct for a possible small lower sensitivity in the central planes (typically observed when 2D SSS is used), because only minor changes in sensitivity over the planes were observed (0.5 ± 0.4 % on average, maximum 4.0 %).

### Scatter Correction

As mentioned above, head motion can introduce reconstruction artefacts due to incorrect scatter scaling estimation. In the case of a mismatch between emission and transmission data (or incorrect segmentation of the skin or nose as air when using a maximum-a-posteriori reconstruction algorithm), real emission lines of responses (LORs) can be misused for scatter scaling resulting in an overestimation of scatter. In order to compensate for small levels of patient motion, ACF-margins of 0, 2, 4, 6, 8, 10, 12 and 14 voxels (*i.e*. up to 17 mm) were applied prior to scatter correction. The placement of this ACF-margin is based on a threshold on ACF voxels (by default 1.03) to determine non-attenuated sinogram bins (*i.e*. lines of responses; LORs) outside the head (*i.e*. the background) that are pure scatter (Fig. 1). ACF-margins were used only to estimate the scatter scaling, *i.e*. they were not used for attenuation correction. Two types of scatter correction methods were used, *i.e*. 2D and 3D SSS [9]. For comparison purposes, 3D SSS was applied to all datasets, whereas 2D SSS was only applied to datasets with a 0- and 10-mm (8 voxels) ACF-margin, respectively.

### Assessment of Patient Motion

The levels of patient motion that occurred during emission scan was assessed using the motion QC tool [6] that is available in the most recent version of the HRRT Software (version 1.3). This tool makes use of the Automated Image Registration software package (AIR version 5, University of California, Los Angeles) to estimate the motion between a summed image of the first minute of the emission scan and the images of the other emission time frames. Note that this method is unable to detect motion in the first 60 s of the emission scan. To assess the levels of patient motion between transmission and emission data, the μ-map and the image of the last time frame of the emission scan were co-registered using a rigid registration method (VINCI software version 4.23, Max Planck Institute for Neurological Research, Cologne, Germany) [16]. The levels of patient motion per subject and per time frame were calculated as $\sqrt{\varDelta {x}^2+\varDelta {y}^2+\varDelta {z}^2}$, where Δ*x*, Δ*y* and Δ*z* are the displacements in the *x*, *y* and *z* direction, respectively.

### Pharmacokinetic Analysis

For each subject, the MR image was co-registered automatically onto the corresponding PET image (summed image from 15 to 60 min) using a rigid registration method (VINCI software version 4.23, Max Planck Institute for Neurological Research, Cologne, Germany) [16]. Prior to pharmacokinetic analysis, VOIs were generated using PVElab (version 2012, Neurobiology Research Unit, Copenhagen, Denmark) [17] in combination with the Hammers template [18]. In addition, statistical parametrical mapping (SPM) (version 8, Institute of Neurology, London, UK) was used to obtain segmentations of grey matter, white matter and extracellular fluid from the MR images. Several VOIs were selected depending on the uptake pattern of the tracer. For the (*R*)-[^11^C]verapamil studies, selected VOIs were the hippocampus, thalamus, striatum, amygdala, parahippocampus, fusiform gyrus and cerebellum. The latter four regions clearly showed artefacts caused by inaccurate scatter scaling. For [^11^C]raclopride studies, the cerebellum, thalamus and striatum were selected, whilst the frontal lobe, thalamus, striatum and brainstem (including pons) were selected for [^11^C]flumazenil studies. Next, time activity curves (TACs) were generated for each VOI.

Metabolite-corrected input functions were derived by processing both data from the on-line blood sampler and manual arterial blood samples. More details can be found in [19] for (*R*)-[^11^C]verapamil, in [20] for [^11^C]raclopride and in [21] for [^11^C]flumazenil. Metabolite-corrected input functions and grey matter TACs were then used to derive the volume of distribution (*V*_T_) for each VOI. A single-tissue compartment plasma-input model with blood volume fraction correction was used for (*R*)-[^11^C]verapamil and [^11^C]flumazenil [22, 23], whilst a reversible two-tissue compartment plasma-input model with blood volume fraction parameter was used for [^11^C]raclopride [24].

### Quantitative Accuracy Assessment

Reconstructed images obtained with different ACF-margins were compared with those obtained without an ACF-margin, using 3D SSS during the reconstruction process. In addition, reconstructed images obtained using 3D SSS were compared with those obtained using 2D SSS, using a 10-mm ACF-margin during the reconstruction process. A Wilcoxon signed rank test (SPSS version 20, Chicago, IL, USA) was used to verify whether an ACF-margin and/or 3D SSS had a significant impact on *V*_T_ values in several different regions when compared with no or less ACF-margin and/or 2D SSS. *P* values were considered statistically significant when they were lower than 0.05.

## Results

### Assessment of Patient Motion

Table 1 reports the levels of patient motion per subject, either observed between transmission and emission scans or observed during the emission scan. There was no statistically significant difference between patient motion observed during the emission scans of different tracer studies as determined by one-way ANOVA (*F*(2,12) = 0.96, *p* = 0.41 and *F*(2,12) = 2.38, *p* = 0.14 for the average and maximum levels of patient motion over all time frames, respectively), but there was a statistically significant difference in patient motion observed between transmission and emission scans of different tracer studies (*F*(2,12) = 5.64, *p* = 0.02). A Tukey post hoc test revealed that the patient motion observed during (*R*)-[^11^C]verapamil studies was statistically significantly higher (4.8 ± 0.9 mm, *p* = 0.03) compared to that observed for [^11^C]raclopride and [^11^C]flumazenil studies (2.9 ± 1.0 and 2.9 ± 1.1 mm, respectively). There were no statistically significant differences between patient motion observed during [^11^C]raclopride and [^11^C]flumazenil studies (*p* = 1.00).

**Table 1:** Amount of patient motion per subject (in mm)

	Tracer	Subject					Mean over all subjects
		1	2	3	4	5	
Average motion during emission scan over all time frames	(*R*)-[^11^C]verapamil	1.8	2.1	3.4	2.2	5.2	3.0
	[^11^C]raclopride	3.1	2.6	1.6	4.5	1.7	2.7
	[^11^C]flumazenil	2.9	2.3	0.9	1.5	2.4	2.0
Maximum motion during emission scan over all time frames	(*R*)-[^11^C]verapamil	4.2	5.9	5.2	4.5	7.7	5.5
	[^11^C]raclopride	10.0	5.2	3.7	7.7	2.7	5.9
	[^11^C]flumazenil	4.2	2.7	2.2	2.9	4.6	3.3
Motion between transmission and emission data	(*R*)-[^11^C]verapamil	4.2	5.9	3.8	4.5	5.5	4.8
	[^11^C]raclopride	2.0	4.5	3.3	2.1	2.7	2.9
	[^11^C]flumazenil	3.2	2.2	1.7	2.9	4.6	2.9

### Impact of an ACF-Margin Prior to Scatter Scaling Estimation on *V*_T_

**Fig. 2:**
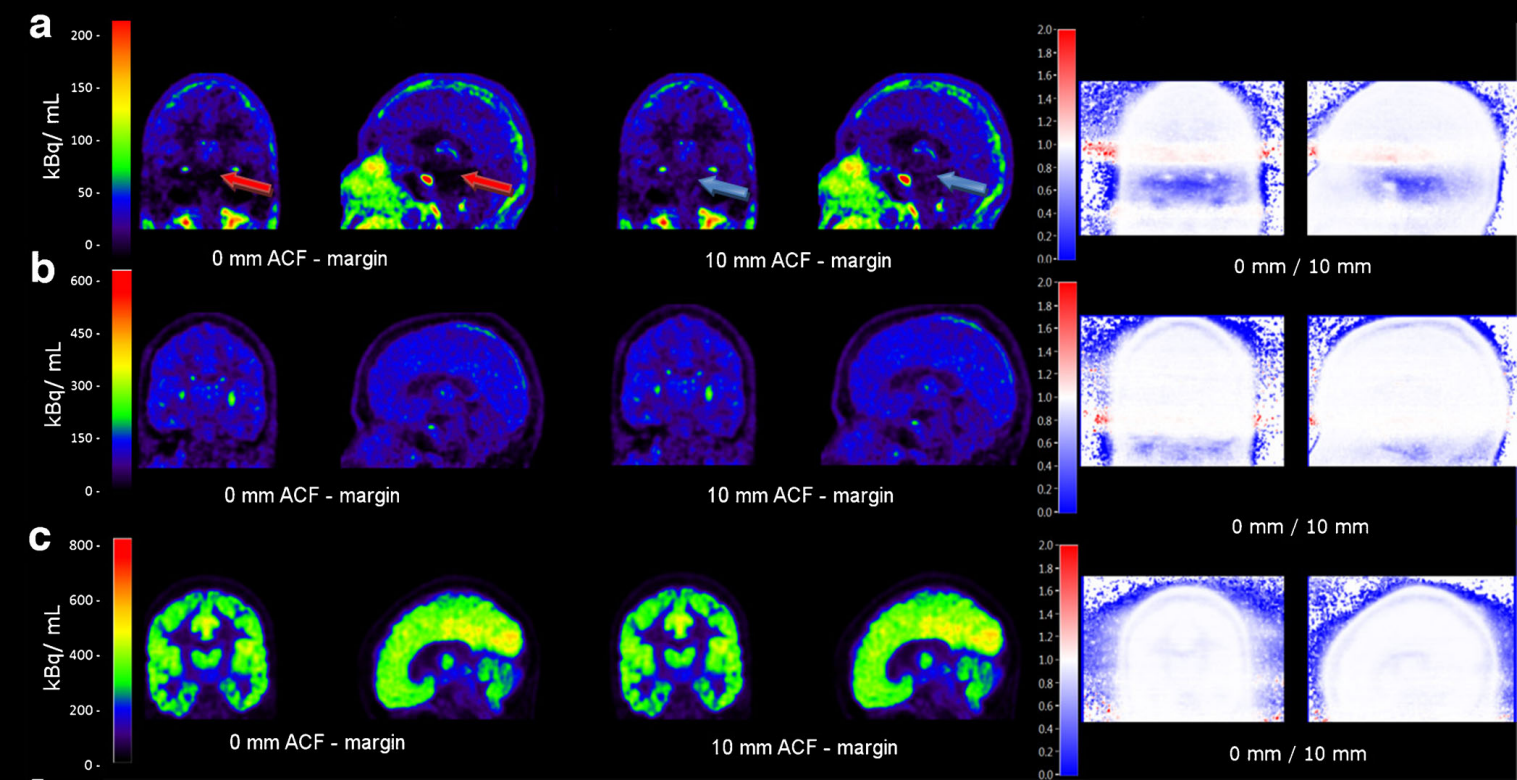
The effects of different ACF-margins prior to scatter scaling estimation on reconstructed activity concentrations (summed from frame 5 to the last frame), illustrated for typical **a** (*R*)-[^11^C]verapamil, **b** [^11^C]raclopride and **c** [^11^C]flumazenil scans in coronal (*left panel*) and sagittal views (*middle panel*). The ratio images (*right panel*) show the ratios of reconstructed activity concentration images without an ACF-margin to those obtained using a 10-mm ACF-margin. The *red arrows* show an area where a prominent artefact caused by a mismatch between emission and transmission data was located. The *blue arrows* indicate the same area. A significant increment in reconstructed activity concentrations was observed when a 10-mm ACF-margin was applied.

**Fig. 3:**
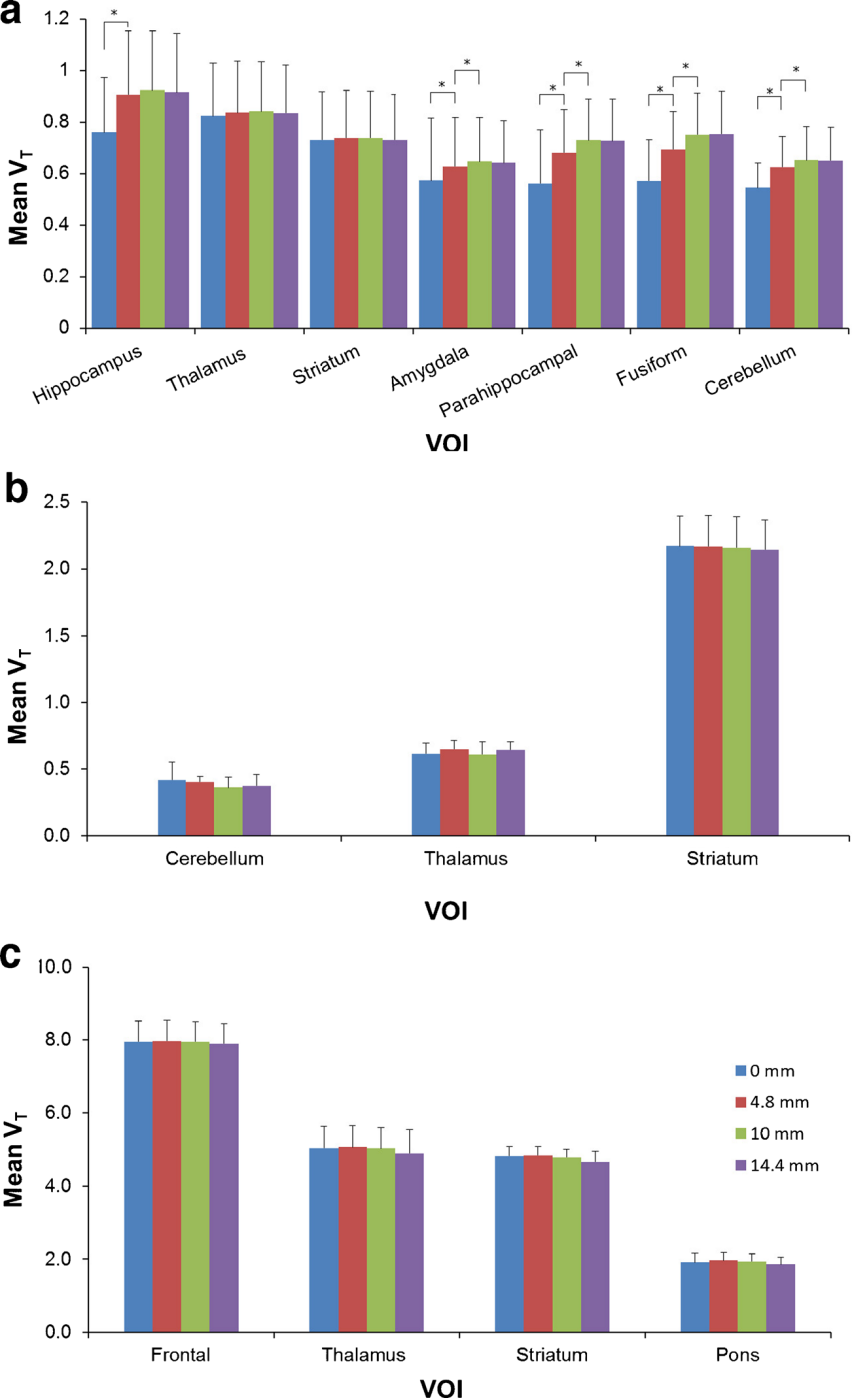
Bar plots illustrating the effects of various ACF-margins prior to scatter scaling estimation on mean *V*_T_ (pooled over five subjects), together with SD, of selected VOIs for **a** (*R*)-[^11^C]verapamil, **b** [^11^C]raclopride and **c** [^11^C]flumazenil. *Stars* indicate statistically significant differences in *V*_T_.

Figure 2 shows coronal and sagittal reconstructed activity concentration images of three typical subjects, one for each tracer. Images were reconstructed using both no ACF-margin and a 10-mm ACF-margin (8 voxels) prior to scatter scaling estimation using 3D SSS. Three out of five (*R*)-[^11^C]verapamil studies showed prominent artefacts in the lower part of the brain with a difference in reconstructed activity concentrations of up to 43 % between no ACF-margin and a 10-mm ACF-margin (8 voxels). When using an ACF-margin of 10 mm (8 voxels) or more, artefacts were no longer visible with higher reconstructed activity concentrations in the artefact areas (Fig. 2). Moreover, for both [^11^C]raclopride and [^11^C]flumazenil studies, all visually apparent artefacts disappeared when an ACF-margin was applied. Figure 3 shows mean *V*_T_ (±SD) over all subjects for different VOIs per tracer. For (*R*)-[^11^C]verapamil, increasing the ACF-margin from 4.8 (4 voxels) to 10 mm (8 voxels) resulted in a significant increase in *V*_T_ of about 24 % for those VOIs that were located in or near the prominent artefact (*i.e*. amygdala, parahippocampal gyrus, fusiform gyrus and cerebellum; *p* < 0.05). On the other hand, there was no further change in *V*_T_ (*p* > 0.05) when the ACF-margin was increased from 10 to 12 mm or more.

For [^11^C]raclopride, ACF-margins did not show significant changes for any of the regions regardless how much margin was applied (change in *V*_T_ was less than 5 % on average; *p* > 0.05). The same effect was seen for [^11^C]flumazenil, where changes in *V*_T_ were less than 3 %, on average, for all VOIs.

### Impact of 2D SSS *Versus* 3D SSS on *V*_T_

**Fig. 4:**
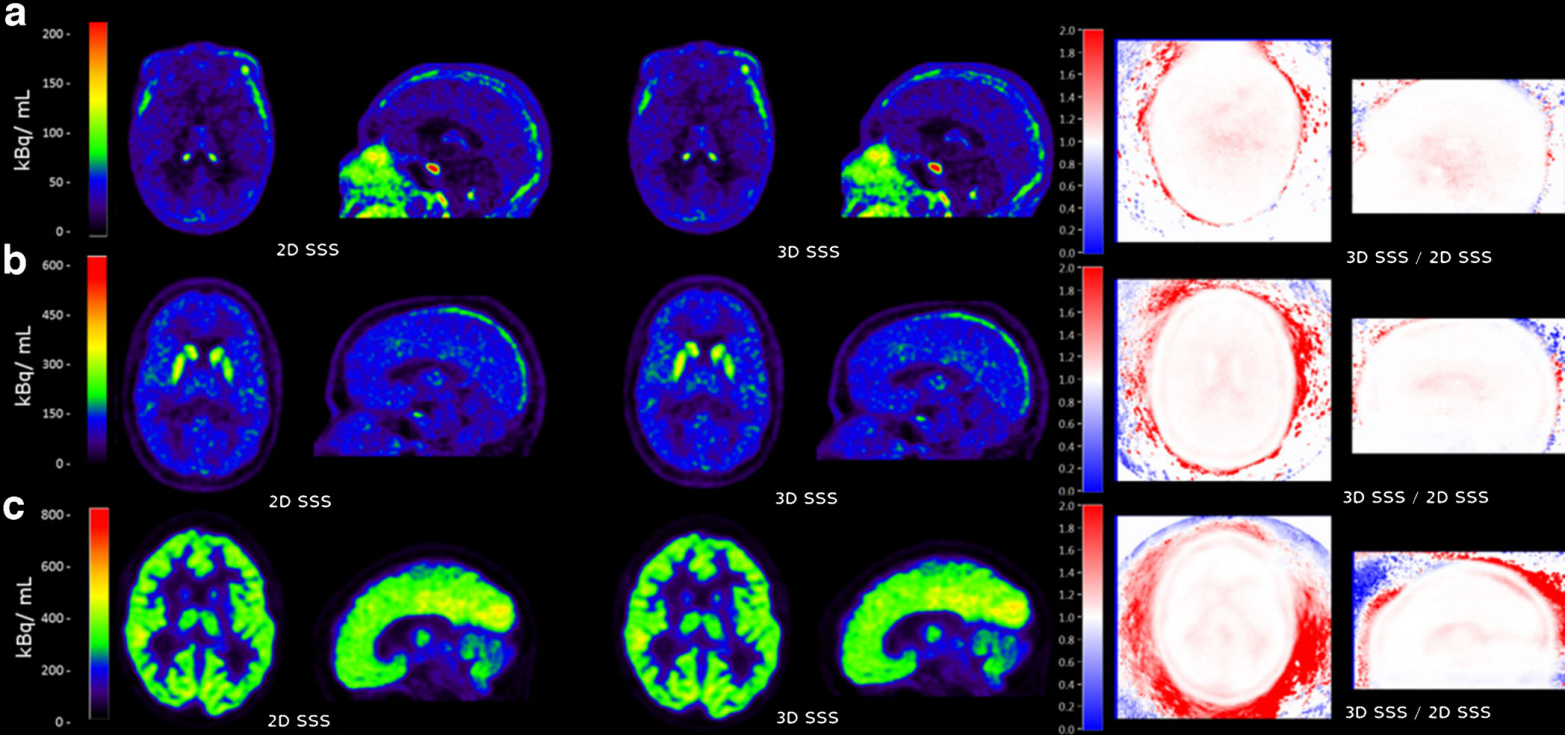
Effects of 2D *versus* 3D SSS on reconstructed activity concentrations (summed from frame 5 to the last frame), illustrated for typical **a** (*R*)-[^11^C]verapamil, **b** [^11^C]raclopride and **c** [^11^C]flumazenil scans in transaxial and sagittal views (*left and middle panels*, respectively). The *images on the right* show the ratios of reconstructed activity concentrations using 3D SSS to those obtained using 2D SSS.

**Fig. 5:**
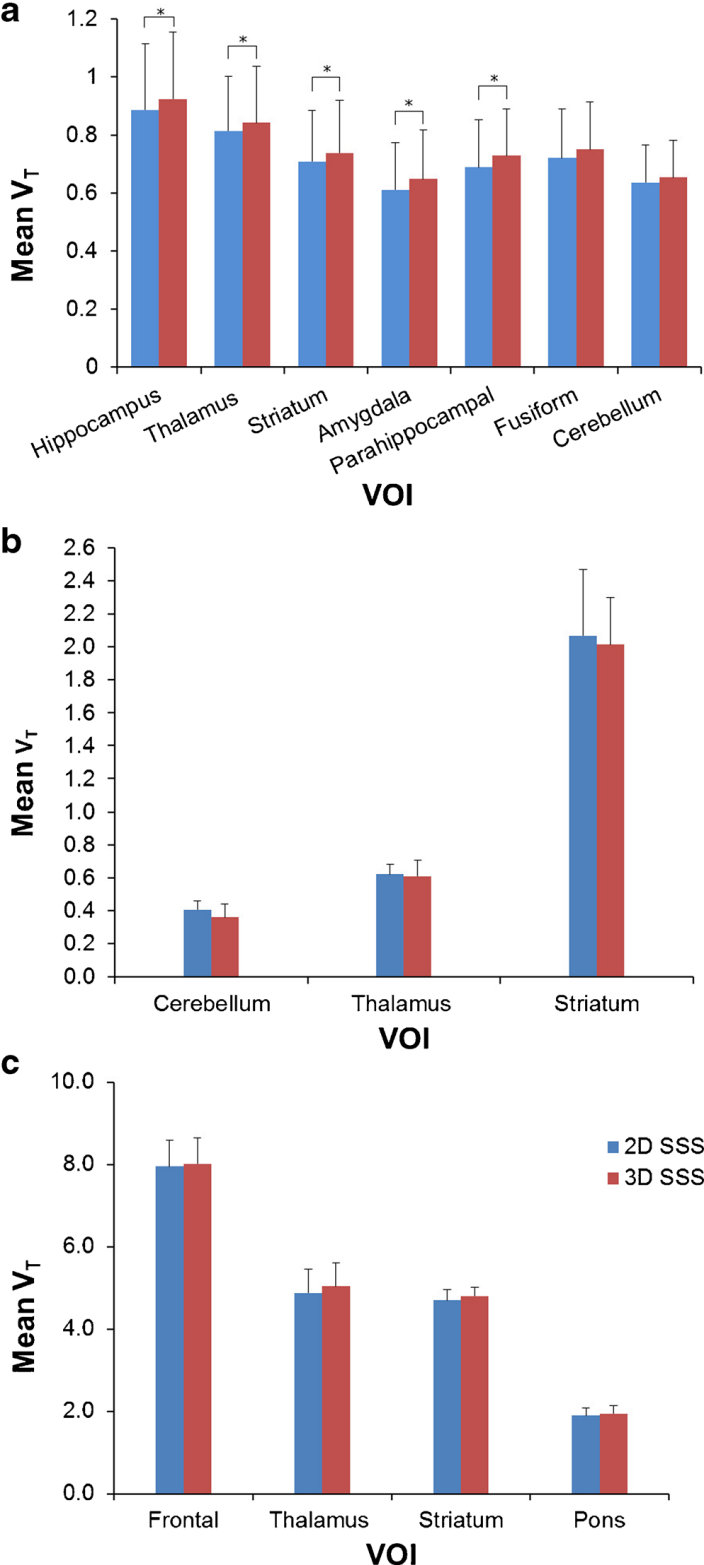
Bar plots illustrating the effects of 2D and 3D SSS on mean *V*_T_ (pooled over five subjects), together with SD, of selected VOIs for **a** (*R*)-[^11^C]verapamil, **b** [^11^C]raclopride and **c** [^11^C]flumazenil. *Stars* indicate statistically significant differences in *V*_T_.

Figure 4 shows transaxial and sagittal reconstructed activity concentration images of three typical subjects, one for each tracer, reconstructed using 2D SSS and 3D SSS scatter correction and a 10-mm ACF-margins. For (*R*)-[^11^C]verapamil, all subjects showed 7 ± 5 % higher reconstructed activity concentrations for 3D SSS than for 2D SSS in the lower part of the brain. Figure 5 shows the effects of 2D SSS and 3D SSS over all subjects for various VOIs. For (*R*)-[^11^C]verapamil, *V*_T_ values increased 4 % on average (range 3 to 6 %) for all VOIs. For the hippocampus, thalamus, striatum, amygdala and parahippocampal gyrus, this increment in *V*_T_ was statistically significant (*p* < 0.05). For [^11^C]raclopride, no statistically significant differences were observed between *V*_T_ obtained using 3D SSS and 2D SSS (*p* > 0.05). There was a small increase of 3 ± 1 % in [^11^C]flumazenil *V*_T_ values when using 3D SSS rather than 2D SSS, but this increase was not statistically significant (*p* > 0.05).

## Discussion

The HRRT scanner is a high-resolution PET system that can visualize tracer distributions in the human brain [1]. When quantifying tracer uptake, there are several factors that can affect quantitative accuracy [7, 25]. One of these factors is patient motion. As previously reported by Anton-Rodriguez *et al*. [6], we occasionally observed a reduction of *V*_T_ in (*R*)-[^11^C]verapamil studies that were caused by a patient motion-induced overestimation of scatter scaling factors. Previous (*R*)-[^11^C]verapamil studies [7, 19, 22] showed a fairly uniform distribution of *V*_T_ values in grey matter areas over the human brain. When patient motion occurs between transmission and emission data, the observed distribution of *V*_T_ becomes less uniform and also deviates from those seen in other (*R*)-[^11^C]verapamil brain studies performed on the ECAT EXACT HR+ (CTI/Siemens, Knoxville, TN, USA) [7], a PET scanner that has been extensively studied for its quantitative accuracy and has been widely used for brain PET studies. By applying an ACF-margin, the distribution of *V*_T_ values becomes more uniform and gets more in line with those observed on the ECAT EXACT HR+ images. We therefore believe that the observed reduction of *V*_T_, caused by a patient motion-induced overestimation of scatter scaling factors, is an artefact.

### Effects of an ACF-Margin on Quantitative Accuracy

In order to compensate for a patient motion-induced mismatch between transmission and emission scans that would lead to an overestimation of scatter scaling factors, a margin was applied to the ACF sinogram prior to the estimation of these scatter scaling factor. The present data illustrate that, by applying an ACF-margin prior to scatter scaling factor estimation, artefacts visually disappeared from the reconstructed images of (*R*)-[^11^C]verapamil studies affected by patient motion. A 10-mm ACF-margin resulted in a statistically significant change in *V*_T_ of up to 24 % in those brain regions where the artefacts were present. When the ACF-margin was larger than 10 mm, no further change in *V*_T_ was seen. This suggests that a 10-mm ACF-margin is sufficient to compensate scatter scaling problems for the levels of patient motion as observed in this study (up to 6 mm) and thus may provide a properly fitted cutoff value of the scatter tail profile as described by Anton-Rodriguez *et al*. [6]. In addition, use of an ACF-margin did not significantly affect *V*_T_ for [^11^C]raclopride and [^11^C]flumazenil. This was expected, since the level of patient motion between transmission and emission was significantly lower for the [^11^C]raclopride and [^11^C]flumazenil studies when compared to the (*R*)-[^11^C]verapamil studies, and (*R*)-[^11^C]verapamil, compared to [^11^C]raclopride and [^11^C]flumazenil, shows higher levels of uptake in or near the outer contour of the patient relative to the levels of uptake in the centre of the brain. This suggests that an ACF-margin can be safely applied for most tracer studies without negatively affecting quantitative accuracy. The method proposed in this study can easily be applied during reconstruction and does not require any additional reconstruction time. In short, it provides an effective way to correct for scatter scaling factor artefacts that are caused by a mismatch between transmission and emission scans due to patient motion. Nevertheless, for large magnitudes of patient motion (>6 mm), not only the scatter scaling estimation will be affected but also tracer uptake, especially for smaller brain structures, due to motion-induced blurring as well as (misaligned) inaccurate attenuation correction [12]. Other methods, such as the one proposed by Anton-Rodriguez *et al*. [6] or Mourik *et al*. [12], should be applied to improve the accuracy of the attenuation correction and to correct for the effects from patient motion during a dynamic (60 min or more) PET study, respectively.

Note, however, that the optimal ACF-margin derived in this study (10 mm) might be study dependent and might not be optimal for different voxel sizes and other fields of view (*e.g*. whole-body imaging). Alternatively, in future applications, the optimal ACF-margin might be determined iteratively (*i.e*. the ACF-margin could be increased until there is no change in the estimated scatter fraction) as the computational cost is relatively small.

### Effects of 2D *Versus* 3D SSS on Quantitative Accuracy

As a P-gp substrate, (*R*)-[^11^C]verapamil is a tracer with low brain uptake, and quantification of its uptake is expected to be more challenging than for [^11^C]raclopride and [^11^C]flumazenil [7]. The present study showed a small, but significant, change in (*R*)-[^11^C]verapamil *V*_T_ for regions in the centre of the brain when 3D SSS was rather than 2D SSS, *V*_T_ remained unchanged for [^11^C]raclopride and [^11^C]flumazenil studies. The observed change may be explained by the incorporation of oblique sinogram planes to estimate scatter when compared to 2D SSS that only uses the non-oblique sinogram. These results are consistent with a previous simulation and phantom study [2]. However, for the lower part of the brain, where most of the reference tissues (*e.g*. cerebellum and pons) are located, 3D SSS might still be inaccurate as it does not compensate for outside FOV scatter, as previously stated in [26]. Due to this outside FOV scatter, 10 % additional scatter was observed in the lower part of the brain when compared with the centre of the brain. This might explain the bias that is still observed in HRRT brain studies when analysed using reference tissue models when compared to brain studies performed on the HR+ [7, 14]. Although the scatter correction methods for both the HRRT and the HR+ scanner are similar, the effect of outside FOV scatter is expected to be lower on the HR+ because a neuro-shield is used during HR+ scanning.

### Future Perspectives

Current and future state-of-the-art clinical PET/CT systems may be equally sensitive to inaccurate scatter scaling factors (*e.g*. due to patient motion) when an SSS algorithm is used, which is similar to that currently in use for the HRRT. Therefore, a relatively easy to implement, fast and low cost method, such as the one presented here, to avoid scatter scaling issues when low levels of patient motion are present is relevant for all PET systems, not just for the HRRT.

There are various alternatives to the current tail-fitting SSS algorithm for estimating scatter. An accurate way to estimate scatter would be to perform a full Monte Carlo (MC) simulation [27]. However, such a method may be computationally expensive. Recently, a new hybrid scatter correction strategy has been proposed for PET/CT studies to overcome scatter scaling issues due to incorrect tail fitting [28]. This method uses SSS to approximate the shape of the scatter contribution but scales it based on a low-count MC simulation. Although initial results are promising [28, 29], MC-SSS is sensitive to outside FOV scatter as well, since it estimates the scatter scaling factor per bed position (*i.e*. not plane by plane). Another promising new alternative for tail fitting, only recommended for time-of-flight (TOF) PET scanners [30], is to estimate the scatter scaling factor as an additional voxel [31], which is updated during each iteration at the same time the activity is updated using a maximum likelihood for activity and attenuation (MLAA) algorithm [32, 33]. MLAA is a promising reconstruction algorithm that could enable future TOF PET/CT and PET/MR scanners to estimate attenuation and scatter without the need for a transmission scan. Unfortunately, the HRRT is not capable of TOF measurements. Nevertheless, full MC simulations to estimate scatter and MC-SSS might be interesting to explore their capacity to further reduce bias that is still observed in HRRT reference tissue model studies.

## Conclusions

When there is a mismatch between transmission and emission scans due to patient motion, applying an ACF-margin results in more reliable scatter scaling factors but does not change (and/or deteriorate) quantification. The effect of applying an ACF-margin is likely tracer dependent and might be more beneficial for tracers such as (*R*)-[^11^C]verapamil, where uptake is prominent in skin tissue (near the outer contour of the patient). 3D SSS, in general, does not significantly alter the quantification of clinical brain PET studies, as it shows a small, yet significant, change in *V*_T_ only for (*R*)-[^11^C]verapamil studies.

In conclusion, use of an ACF-margin can avoid artefacts in reconstructed HRRT images due to motion-induced scatter scaling errors. This method might be of interest for other commercially available PET systems that use the same scatter scaling method.

## References

[1] de Jong HW, van Velden FH, Kloet RW (2007). Performance evaluation of the ECAT HRRT: an LSO-LYSO double layer high resolution, high sensitivity scanner. Phys Med Biol.

[2] Sibomana M, Keller SH, Stute S, Comtat C (2012) Benefits of 3D scatter correction for the HRRT—a large axial FOV PET scanner. IEEE Nuclear Science Symposium Conference Record (NSS/MIC) 2954–2957

[3] Keller SH, Svarer C, Sibomana M (2013). Attenuation correction for the HRRT PET-scanner using transmission scatter correction and total variation regularization. IEEE Trans Med Imaging.

[4] Walker MD, Asselin MC, Julyan PJ (2011). Bias in iterative reconstruction of low-statistics PET data: benefits of a resolution model. Phys Med Biol.

[5] Ju-Chieh C, Blinder S, Rahmim A, Sossi V (2010). A scatter calibration technique for dynamic brain imaging in high resolution PET. IEEE Trans Nucl Sci.

[6] Anton-Rodriguez JM, Sibomana M, Walker MD et al (2010) Investigation of motion induced errors in scatter correction for the HRRT brain scanner. IEEE Nuclear Science Symposium Conference Record (NSS/MIC) 2935–2940

[7] van Velden FH, Mansor SM, van Assema DM (2015). Comparison of HRRT and HR+ scanners for quantitative (R)-[^11^C]verapamil, [^11^C]raclopride and [^11^C]flumazenil brain studies. Mol Imaging Biol.

[8] Sossi V, De Jong HW, Barker WC et al (2005) The second generation HRRT—a multi-centre scanner performance investigation. IEEE Nuclear Science Symposium Conference Record (NSS/MIC) 4:2195–2199

[9] Watson CC (2000). New, faster, image-based scatter correction for 3D PET. IEEE Trans Nucl Sci.

[10] Hong I, Burbar Z, Michel C (2011) True 3D iterative scatter correction for small bore long axial FOV scanner. IEEE Nuclear Science Symposium Conference Record (NSS/MIC) 3736–3738

[11] Keller SH, Sibomana M, Olesen OV (2012). Methods for motion correction evaluation using ^18^F-FDG human brain scans on a high-resolution PET scanner. J Nucl Med.

[12] Mourik JE, Lubberink M, van Velden FH (2009). Off-line motion correction methods for multi-frame PET data. Eur J Nucl Med Mol Imaging.

[13] Boellaard R, van Lingen A, van Balen SC (2001). Characteristics of a new fully programmable blood sampling device for monitoring blood radioactivity during PET. Eur J Nucl Med.

[14] van Velden FH, Kloet RW, van Berckel BN (2009). HRRT *versus* HR+ human brain PET studies: an interscanner test-retest study. J Nucl Med.

[15] Sureau FC, Reader AJ, Comtat C (2008). Impact of image-space resolution modeling for studies with the high-resolution research tomograph. J Nucl Med.

[16] Cizek J, Herholz K, Vollmar S (2004). Fast and robust registration of PET and MR images of human brain. Neuroimage.

[17] Svarer C, Madsen K, Hasselbalch SG (2005). MR-based automatic delineation of volumes of interest in human brain PET images using probability maps. Neuroimage.

[18] Hammers A, Koepp MJ, Free SL (2002). Implementation and application of a brain template for multiple volumes of interest. Hum Brain Mapp.

[19] van Assema DM, Lubberink M, Boellaard R (2012). Reproducibility of quantitative (R)-[^11^C]verapamil studies. EJNMMI Res.

[20] Schuit R, Luurtsema G, Greuter H (2007). Intravenous amphetamine administration does not affect metabolism of [^11^C]raclopride. J Label Compd Radiopharm.

[21] Liefaard LC, Ploeger BA, Molthoff CF (2009). Changes in GABAA receptor properties in amygdala kindled animals: in vivo studies using [11C]flumazenil and positron emission tomography. Epilepsia.

[22] Lubberink M, Luurtsema G, van Berckel BN (2007). Evaluation of tracer kinetic models for quantification of P-glycoprotein function using (R)-[11C]verapamil and PET. J Cereb Blood Flow Metab.

[23] Klumpers UM, Veltman DJ, Boellaard R (2008). Comparison of plasma input and reference tissue models for analysing [(11)C]flumazenil studies. J Cereb Blood Flow Metab.

[24] Lammertsma AA, Bench CJ, Hume SP (1996). Comparison of methods for analysis of clinical [^11^C]raclopride studies. J Cereb Blood Flow Metab.

[25] Walker MD, Sossi V (2015). Commentary: an eye on PET quantification. Mol Imaging Biol.

[26] Kloet RW, De Jong HW, van Velden FH et al (2006) Influence of outside field of view activity on the quality of high resolution research tomograph (HRRT) brain studies. IEEE Nuclear Science Symposium Conference Record (NSS/MIC) 3369–3371

[27] Levin CS, Dahlbom M, Hoffman EJ (1995). A Monte Carlo correction for the effect of Compton scattering in 3-D PET brain imaging. IEEE Trans Nucl Sci.

[28] Jinghan Y, Xiyun S, Zhiqiang H (2014) Scatter correction with combined single-scatter simulation and Monte Carlo simulation for 3D PET. IEEE Nuclear Science Symposium Conference Record (NSS/MIC)

[29] Jarmo T, Jarkko J, Jani L et al (2014) Comparison of single-scatter simulation and Monte Carlo single-scatter simulation on Philips Ingenuity TF PET/MR. IEEE Nuclear Science Symposium Conference Record (NSS/MIC)

[30] Boellaard R, Hofman MB, Hoekstra OS, Lammertsma AA (2014). Accurate PET/MR quantification using time of flight MLAA image reconstruction. Mol Imaging Biol.

[31] Defrise M, Salvo K, Rezaei A et al (2014) ML estimation of the scatter scaling in TOF PET. IEEE Nuclear Science Symposium Conference Record (NSS/MIC)

[32] Nuyts J, Rezaei A, Defrise M (2012) ML-reconstruction for TOF-PET with simultaneous estimation of the attenuation factors. IEEE Nuclear Science Symposium Conference Record (NSS/MIC) 2147–2149 10.1109/TMI.2014.2318175 24760903

[33] Nuyts J, Dupont P, Stroobants S (1999). Simultaneous maximum a posteriori reconstruction of attenuation and activity distributions from emission sinograms. IEEE Trans Med Imaging.

